# Sleep and Autonomic Manifestations in Parkinson’s Disease Complicated With Probable Rapid Eye Movement Sleep Behavior Disorder

**DOI:** 10.3389/fnins.2022.874349

**Published:** 2022-04-08

**Authors:** Hiroaki Fujita, Tomohiko Shiina, Hirotaka Sakuramoto, Narihiro Nozawa, Keitaro Ogaki, Keisuke Suzuki

**Affiliations:** Department of Neurology, Dokkyo Medical University, Tochigi, Japan

**Keywords:** Parkinson’s disease, rapid eye movement sleep behavior disorder, PDSS-2, visual hallucination, SCOPA-AUT, lower urinary tract symptoms

## Abstract

Patients with Parkinson’s disease (PD) complicated with rapid eye movement sleep behavior disorder (RBD) present with distinct clinical features. The purpose of this study was to determine the clinical features of sleep and autonomic symptoms in PD patients with probable RBD (pRBD). The study included 126 patients with PD. pRBD was defined as having a history of dream-enacting behavior with a total score of 5 or greater on the Japanese version of the RBD Screening Questionnaire (RBDSQ-J). The Parkinson’s Disease Sleep Scale-2 (PDSS-2) was used to evaluate sleep disturbances. Scales for Outcomes in Parkinson’s Disease-Autonomic dysfunction (SCOPA-AUT) were used to evaluate autonomic symptoms. Clinical assessments included disease severity, motor symptoms, olfaction, depression, cognitive function, levodopa equivalent dose (LED), and cardiac metaiodobenzylguanidine (MIBG) scintigraphy. Correlations between RBDSQ-J total scores and clinical variables were analyzed. Compared to PD patients without pRBD, PD patients with pRBD showed severe hyposmia, severe sleep-related symptoms, severe dysautonomia, and more reduced cardiac MIBG scintigraphy. Within the PDSS-2, the “PD symptoms at night” domain was significantly more severe in PD patients with pRBD. Within the SCOPA-AUT, the “urinary” and “cardiovascular” domains were significantly higher in PD patients with pRBD. In correlation analyses, RBDSQ-J total scores were positively correlated with PDSS-2 total scores, “PD symptoms at night” and “disturbed sleep” domains, Epworth Sleepiness Scale scores, SCOPA-AUT total scores, “urinary,” “cardiovascular,” and “thermo” domain scores, and LED. RBDSQ-J total scores were negatively correlated with cardiac MIBG scintigraphy uptake. Binary logistic regression analysis showed that PDSS-2 subitem 7 (distressing hallucinations) and SCOPA-AUT subitem 11 (weak stream of urine) were significant determinants for pRBD. Our study showed that PD patients with pRBD had characteristic sleep and autonomic symptoms.

## Introduction

Parkinson’s disease (PD) is the most common neurodegenerative movement disorder (Balestrino and Schapira, 2020) and is associated with Lewy body pathology in the central and peripheral nervous systems. Although PD is characterized by striking motor symptoms such as bradykinesia, tremor, and rigidity, numerous non-motor symptoms appear, which often precede motor symptoms. Rapid eye movement sleep behavior disorder (RBD), a non-motor symptom, is a parasomnia characterized by dream enactment behavior and loss of normal atonia during rapid eye movement (REM) sleep. RBD is categorized into isolated RBD and secondary RBD, and the former has received much attention as a prodromal condition of PD because of its high phenoconversion rate (Postuma et al., 2019). It has also been reported that PD patients with RBD tend to show distinct clinical features compared to PD patients without RBD (Suzuki et al., 2017; Iijima et al., 2021), and RBD is considered a predictor of the diffuse malignant PD subtype, which presents rapid progression and resistance to medication (Fereshtehnejad et al., 2015). Sleep-related symptoms and autonomic symptoms are also major non-motor symptoms in PD that affect quality of life (Suzuki et al., 2013; Merola et al., 2018). Previously, a few reports investigated sleep and autonomic symptoms in PD patients with RBD (Iranzo et al., 2009; Nomura et al., 2013; Kim et al., 2016). However, there has been little research on what types of sleep problems and autonomic symptoms are involved in PD with RBD. The aim of this study was to comprehensively assess the clinical features of PD patients with probable RBD (pRBD), especially focusing on sleep and autonomic manifestations, which may be useful to consider the underlying pathophysiology of PD complicated with RBD.

## Materials and Methods

This cross-sectional study was performed in accordance with the Declaration of Helsinki and approved by the Institutional Review Board of Dokkyo Medical University. All participants provided written informed consent.

### Subjects

In this study, from April 2018 to March 2021, 126 consecutive patients with PD in whom pRBD was assessed. The diagnosis of PD was made based on the Movement Disorder Society-PD criteria (Postuma et al., 2015b). All participants were interviewed according to the International Restless Legs Syndrome Study Group criteria (Allen et al., 2003) to rule out restless legs syndrome comorbidity. No individuals were taking antidepressants. A total of 80.1% of the patients underwent a dopamine transporter scan to assess presynaptic dopaminergic dysfunction. All patients were interviewed about their history of dream-enacting behavior according to the clinical International Classification of Sleep Disorders, revised (ICSD-R), minimal diagnostic criteria (Vignatelli et al., 2005). pRBD was defined when patients had a history of dream behavior and scored ≥5 points on the Japanese version of the RBD Screening Questionnaire (RBDSQ-J). Family members or caregivers were allowed to help the PD patients fill out their questionnaires.

### Clinical Assessments

Disease severity was rated using the Hoehn and Yahr (HY) stage (Hoehn and Yahr, 1967). Motor symptoms were assessed by the Movement Disorder Society Unified Parkinson’s Disease Rating Scale (MDS-UPDRS) part III. Cognitive function was assessed by the Japanese version of the Mini-Mental State Examination (MMSE) (Mori and Yamadori, 1985; Ideno et al., 2012). Levodopa equivalent doses (LEDs) were calculated based on previously described methods (Schade et al., 2020). Olfactory function was assessed by Open Essence (Wako, Japan), a card-type odor identification test (Saito et al., 2006). Sleepiness during the daytime was measured by the Japanese version of the Epworth Sleepiness Scale (ESS) (Takegami et al., 2009). Sleep symptoms were assessed by the Japanese version of the Parkinson’s Disease Sleep Scale-2 (PDSS-2), which consists of 15 PD-related nocturnal problems (Suzuki et al., 2012). The PDSS-2 was divided into three domains: “motor symptoms at night” (items 4–6, 12, and 13), “PD symptoms at night” (items 7, 9–11, and 15), and “disturbed sleep” (items 1–3, 8, and 14). We excluded the patients identified *via* interview with the patients and their bed partners as habitual snorers, defined as a snoring frequency of ≥2 days per week. Autonomic symptoms were assessed using the Japanese version of the Scales for Outcomes in Parkinson’s Disease-Autonomic dysfunction (SCOPA-AUT) (Matsushima et al., 2014). The SCOPA-AUT consists of 25 subitems regarding autonomic symptoms related to PD, which are divided into seven domains [gastrointestinal, items 1–7; urinary, items 8–13; cardiovascular, items 14–16, thermo, items 17, 18, 20, and 21; pupillomotor, item 19; sexual function (male), items 22 and 23; and sexual function (female), items 24 and 25]. Depression was assessed by the Beck Depression Inventory-II (BDI-II). Cardiac metaiodobenzylguanidine (MIBG) scintigraphy was conducted to assess the cardiac sympathetic innervation. After the subjects had rested for 15 min in a supine position, 111MBq ^123^I-MIBG (Fujifilm RI Pharma Co., Tokyo, Japan) was intravenously injected. Chest SPECT and planar images were performed using a gamma camera at 15 min (early phase) and 4 h (delayed phase) after injection, respectively. The heart-to-mediastinum ratio was calculated by dividing the count density of the left ventricular region of interest (ROI) by that of the mediastinum ROI.

### Statistical Analyses

A Mann–Whitney *U* test was used to compare continuous variables. A Chi-square or Fisher’s exact test was used to compare categorical variables. Spearman rank correlation coefficients were used to assess correlations between RBDSQ-J scores and clinical variables. Binary logistic regression analysis with a forward selection likelihood ratio was performed to determine the contributing factors to PD patients with pRBD. A *P*-value < 0.05 was considered statistically significant. Analyses were performed with SPSS Statistics, Version 27 (IBM SPSS, Tokyo, Japan).

## Results

Among 126 consecutive PD patients, 31 patients (25%) had pRBD complications. The clinical features of PD patients with and without pRBD are shown in Table 1. Age, gender distribution, body mass index, and the rate of comorbidities, such as hypertension, diabetes mellitus, and prostate disease (male), PD disease duration, ratio of *de novo* patients, HY stage, MDS-UPDRS Part III, MMSE, BDI-II, and LED did not show significant differences between the two groups. Dopamine agonists were used in 23 (patch formulation, 13; extended-release formulation; 4 and other, 6) of the 95 PD patients without pRBD and 6 (patch formulation, 1; extended release formulation, 3; other 2) of 31 the PD patients with pRBD. Five and four patients were treated with a monoamine oxidase-B (MAO-B) inhibitor or a catechol-*O*-methyltransferase (COMT) inhibitor, respectively, among the patients without pRBD, and one and two patients were treated with a MAO-B inhibitor or a COMT inhibitor, respectively, among the patients with pRBD. The number of patients administered these medications did not significantly differ between the two groups. Compared to the PD patients without pRBD, the PD patients with pRBD showed severe olfactory dysfunction, severe daytime sleepiness and sleep-related problems assessed by the ESS and PDSS-2, respectively, severe dysautonomia assessed by the SCOPA-AUT, and more reduced uptake in cardiac MIBG scintigraphy in the delayed phase. Table 2 summarizes the comparison of PDSS-2 domains and subitems between the two groups. In PDSS-2, the “PD symptoms at night” domain was significantly higher in the PD patients with pRBD, whereas the domains of “motor symptoms at night” and “disturbed sleep” did not reach significance. Within the PDSS-2 items, item 6 (distressing dreams), item 7 (distressing hallucinations), item 11 (muscle cramps in arms or legs), item 14 (tired/sleepy in the morning), and item 15 (breathing problems/snoring) were more frequent in the PD patients with pRBD. Among the SCOPA-AUT domains, the “urinary” and “cardiovascular” domains were higher in the PD patients with pRBD (Table 3). Within the “urinary” domain, item 10 (feeling of residual urine), item 11 (weak stream of urine), and item 13 (nocturia) showed significant differences between the PD patients with pRBD and those without pRBD (0.68 ± 0.77 vs. 0.35 ± 0.67, *P* = 0.011; 1.07 ± 0.87 vs. 0.51 ± 0.75, *P* = 0.001; 1.93 ± 1.04 vs. 1.45 ± 1.01, *P* = 0.042, respectively). Within the “cardiovascular” domain, item 14 (symptoms at standing up) showed a significant difference between the PD patients with pRBD and those without pRBD (0.76 ± 0.79 vs. 0.32 ± 0.66, *P* < 0.001). In correlation analyses, RBDSQ-J total scores were positively correlated with PDSS-2 total, “PD symptoms at night” and “disturbed sleep” scores, ESS scores, SCOPA-AUT scores in the “urinary,” “cardiovascular,” and “thermo” domains, and LED. RBDSQ-J total scores were negatively correlated with cardiac MIBG scintigraphy uptake (Table 4). Binary logistic regression analysis showed that PDSS-2 subitem 7 (distressing hallucinations) and SCOPA-AUT subitem 11 (weak stream of urine) were significant determinants for pRBD (Table 5).

**TABLE 1:** Comparison of clinical variables between PD patients with pRBD and PD patients without pRBD.

	PD with pRBD (*n* = 31)	PD without pRBD (*n* = 95)	*P*-value
Age (years)	71.3 ± 7.1	68.4 ± 9.5	0.118
Sex (M/F)	17/14	40/55	0.152
Body mass index	23.6 ± 3.8	22.7 ± 3.2	0.194
Hypertension	9	31	0.709
Diabetes mellitus	3	12	0.659
Prostate disease	4/17 (24%)	11/40 (28%)	0.465
Disease duration (years)	4.61 ± 4.66	3.49 ± 3.56	0.264
*De novo*, *n* (%)	14 (45)	42 (44)	0.818
HY stage	2.74 ± 0.97	2.47 ± 0.90	0.250
MDS-UPDRS Part III	31.7 ± 18.2	28.7 ± 15.8	0.526
MMSE	25.5 ± 4.2	26.6 ± 3.1	0.278
BDI-II	14.3 ± 8.9	12.8 ± 8.9	0.371
PDSS-2	16.4 ± 9.2	11.7 ± 7.9	**0.006**
ESS	9.16 ± 5.77	7.21 ± 6.13	**0.037**
SCOPA-AUT	14.2 ± 8.63	10.3 ± 6.98	**0.024**
Olfactory test (OE)	3.13 ± 2.33	4.00 ± 2.17	**0.049**
Cardiac MIBG scintigraphy (H/M ratio)			
Early phase	2.03 ± 0.73	2.20 ± 0.64	0.086
Delay phase	1.69 ± 0.71	2.04 ± 0.96	**0.033**
LED (mg/day)	257.3 ± 267.2	227.1 ± 329.2	0.222
Use of dopamine agonist	6	23	0.577
Use of MAO-B inhibitor	1	5	0.644
Use of COMT inhibitor	2	4	0.635

*P-values < 0.05 are highlighted in bold text.*

*PD, Parkinson’s disease; pRBD, probable rapid eye movement sleep behavior disorder; HY, Hoehn and Yahr; MDS-UPDRS, Movement Disorder Society Unified Parkinson’s Disease Rating Scale; MMSE, Mini-Mental State Examination; BDI-II, Beck Depression Inventory-II; PDSS-2, Parkinson’s Disease Sleep Scale-2; ESS, Epworth Sleepiness Scale; RBDSQ-J, RBD Screening Questionnaire Japanese version; SCOPA-AUT, Scales for Outcomes in Parkinson’s Disease-Autonomic dysfunction; OE, Open Essence; MIBG, metaiodobenzylguanidine; H/M ratio, heart-to-mediastinum ratio; LED, levodopa equivalent dose; MAO-B, monoamine oxidase-B; COMT, catechol-O-methyltransferase.*

**TABLE 2:** Comparison of PDSS-2 subitems between PD patients with and without pRBD.

Items	PD with pRBD	PD without pRBD	*P*-value
**Disturbed sleep**	8.84 ± 4.35	7.41 ± 3.84	0.058
1. Bad sleep quality	1.53 ± 1.28	1.12 ± 1.26	0.091
2. Difficulty falling asleep	1.27 ± 1.23	1.02 ± 1.14	0.319
3. Difficulty staying asleep	2.27 ± 1.41	2.03 ± 1.25	0.367
8. Get up to pass urine	2.47 ± 1.76	2.31 ± 1.29	0.810
14. Tired/sleepy in the morning	1.60 ± 1.00	1.04 ± 1.10	**0.008**
**Motor symptoms at night**	3.68 ± 3.81	2.35 ± 3.02	0.075
4. Restlessness of arms or legs	0.87 ± 0.97	0.77 ± 1.05	0.459
5. Urge to move arms or legs	0.77 ± 1.01	0.50 ± 0.80	0.177
6. Distressing dreams	0.97 ± 0.93	0.32 ± 0.61	**<0.001**
12. Painful posturing in morning	0.50 ± 0.77	0.28 ± 0.68	0.052
13. Tremor on walking	0.70 ± 0.87	0.51 ± 0.87	0.167
**PD symptoms at night**	3.90 ± 3.45	1.94 ± 2.66	**0.002**
7. Distressing hallucination	0.93 ± 1.17	0.12 ± 0.32	**<0.001**
9. Uncomfortable and immobile	0.90 ± 1.03	0.60 ± 0.91	0.117
10. Pain in arms or legs	0.67 ± 0.80	0.47 ± 0.80	0.139
11. Muscle cramps in arms or legs	0.77 ± 0.82	0.46 ± 0.80	**0.026**
15. Breathing problems/snoring	0.77 ± 0.86	0.32 ± 0.61	**0.004**

*P-values < 0.05 are highlighted in bold text.*

*PD, Parkinson’s disease; pRBD, probable rapid eye movement sleep behavior disorder; PDSS-2, Parkinson’s Disease Sleep Scale-2.*

**TABLE 3:** Comparison of SCOPA-AUT domains between PD patients with and without pRBD.

	PD with pRBD	PD without pRBD	*P*-value
Gastrointestinal	4.67 ± 3.93	3.09 ± 2.85	0.053
Urinary	5.37 ± 3.28	4.12 ± 2.95	**0.034**
Cardiovascular	1.07 ± 1.17	0.57 ± 1.21	**0.004**
Thermo	0.97 ± 1.61	0.53 ± 0.99	0.173
Pupillomotor	1.07 ± 1.29	0.87 ± 1.27	0.258
Sexual function, male	0.23 ± 0.82	0.33 ± 1.14	0.891
Sexual function, female	0.0 ± 0.0	0.07 ± 0.46	0.417
Total	15.5 ± 9.12	10.3 ± 6.80	**0.024**

*P-values < 0.05 are highlighted in bold text.*

*PD, Parkinson’s disease; pRBD, probable rapid eye movement sleep behavior disorder; SCOPA-AUT, Scales for Outcomes in Parkinson’s Disease-Autonomic dysfunction.*

**TABLE 4:** Correlation analysis between RBDSQ-J total scores and clinical variables.

	RBDSQ-J total score
Age	0.124
Disease duration	0.115
HY stage	0.161
MDS-UPDRS Part III	0.081
MMSE	−0.059
BDI-II	0.128
PDSS-II	0.301**
Motor symptoms at night	0.155
PD symptoms at night	0.305**
Disturbed sleep	0.240**
Item 1. Bad sleep quality	0.147
Item 2. Difficulty falling asleep	0.104
Item 3. Difficulty staying asleep	0.225*
Item 4. Restlessness of arms or legs	0.077
Item 5. Urge to move arms or legs	0.102
Item 6. Distressing dreams	0.321**
Item 7. Distressing hallucination	0.411**
Item 8. Get up to pass urine	0.107
Item 9. Uncomfortable and immobile	0.233**
Item 10. Pain in arms or legs	0.113
Item 11. Muscle cramps in arms or legs	0.192*
Item 12. Painful posturing in morning	0.112
Item 13. Tremor on walking	0.129
Item 14. Tired/sleepy in the morning	0.268**
Item 15. Breathing problems/snoring	0.232**
ESS	0.233**
SCOPA-AUT total	0.208*
Gastrointestinal	0.167
Urinary	0.289**
Cardiovascular	0.188*
Thermo	0.214*
Pupillomotor	0.068
Sexual function, male	0.003
Sexual function, female	0.000
Olfactory test	−0.110
Cardiac MIBG scintigraphy	
Early phase	−0.227*
Delay phase	−0.268**
LED	0.201*

*Spearman rank correlation coefficients.*

*P < 0.05.

**P < 0.01.

*P-values < 0.05 are highlighted in bold text.*

*PD, Parkinson’s disease; HY, Hoehn and Yahr; MDS-UPDRS, Movement Disorder Society Unified Parkinson’s Disease Rating Scale; MMSE, Mini-Mental State Examination; BDI-II, Beck Depression Inventory-II; PDSS-2, Parkinson’s Disease Sleep Scale-2; ESS, Epworth Sleepiness Scale; RBDSQ-J, RBD Screening Questionnaire Japanese version; SCOPA-AUT, Scales for Outcomes in Parkinson’s Disease-Autonomic dysfunction; MIBG, metaiodobenzylguanidine; LED, levodopa equivalent dose.*

**TABLE 5:** Binary logistic regression analysis of clinical variables predictive of probable RBD in PD patients.

	OR (95% CI)	*P*
PDSS-2 subitem 7 (distressing hallucinations)	3.783 (1.712–8.362)	**0.001**
SCOPA-AUT subitem 11 (weak stream of urine)	1.875 (1.075–3.271)	**0.027**

*Using the forward selection likelihood ratio.*

*Independent variables: age, sex, disease duration, MDS-UPDRS III, PDSS-2 subitems 6, 7, 11, 14, and 15, and SCOPA-AUT subitems 10, 11, 13, and 14.*

*P-values < 0.05 are highlighted in bold text.*

*PD, Parkinson’s disease; RBD, rapid eye movement sleep behavior disorder; PDSS-2, Parkinson’s Disease Sleep Scale-2; SCOPA-AUT, Scales for Outcomes in Parkinson’s Disease-Autonomic dysfunction.*

## Discussion

In the present study, we showed that PD patients with pRBD had more severe clinical symptoms and findings, including sleep-related symptoms, autonomic symptoms, hyposmia, and reduced cardiac MIBG scintigraphy, than PD patients without pRBD, which was consistent with previous reports (Postuma et al., 2012; St Louis et al., 2017; Pagano et al., 2018; Nomura et al., 2020). Although motor symptoms were more severe in PD patients with RBD in some previous studies (Suzuki et al., 2017) and MDS-UPDRS part III scores tended to be higher in the PD patients with pRBD in the present study, this difference did not reach significance. RBDSQ-J total scores did not correlate with disease duration, disease severity, motor symptom severity, or cognitive function in the present study.

Regarding sleep-related symptoms, PDSS-2 subitem 7 (distressing hallucinations) was a determinant for pRBD by binary logistic regression analysis. Liu et al. (2017) reported that 20% of PD patients with pRBD had visual hallucinations assessed by PDSS subitem 7, compared to 6.3% in PD patients without pRBD. Pagano et al. (2018) reported that hallucinations assessed by MDS-UPDRS Part I were more severe in PD patients with pRBD. Taken together, it is postulated that RBD and visual hallucinations are closely related. Although the pathogenesis of visual hallucinations in PD with RBD is not fully understood, dysfunction of the pedunculopontine nucleus (PPN), which is compromised in both PD and RBD, is considered to be related to visual hallucinations (Hepp et al., 2013). In addition, a resting-state functional connectivity magnetic resonance imaging study in RBD patients showed reduced connections between lateral geniculate nuclei and the visual association cortex (Geddes et al., 2016). The fact that cholinesterase inhibitors are somewhat effective against visual hallucinations in PD patients supports the hypothesis that the cholinergic nervous network centered in the PPN is involved in the occurrence of visual hallucinations (Burn et al., 2006). Degenerative loss of cholinergic nerves, including the PPN, along with glutamatergic nerves centered in the sublaterodorsal tegmental nucleus are considered to play an important role in the loss of atonia during the REM sleep period (Dauvilliers et al., 2018); therefore, PD complicated by RBD may be more prone to visual hallucinations. Interestingly, in PD patients with nocturnal hallucinations, REM sleep without atonia was frequently observed in close proximity to the onset of hallucinations (Nomura et al., 2003).

Given that PDSS-2 item 14 (tired/sleepy in the morning) and ESS scores, the PD patients with pRBD showed severe daytime sleepiness in the present study. It has been reported that daytime sleepiness in PD is caused not only by a decrease in total sleep time but also by a variety of other factors, including poor sleep quality, circadian rhythm sleep disorders, and disturbances in the ascending arousal system (Feng et al., 2021). In the PDSS-2 “PD symptoms at night” domain, subitems 7 (distressing hallucinations), 11 (muscle cramps in arms or legs), and 15 (breathing problems/snoring) were significantly higher in PD patients with pRBD, and these symptoms may contribute to nocturnal arousal and daytime sleepiness.

Compared to PD patients without pRBD, PD patients with pRBD had severe autonomic symptoms assessed by the SCOPA-AUT, which was consistent with previous reports (Liu et al., 2017; St Louis et al., 2017; Nomura et al., 2020). Among the domains of the SCOPA-AUT, the “urinary” and “cardiovascular” domains were higher in the PD patients with pRBD, and RBDSQ-J total scores had a positive correlation with scores in both domains. Logistic regression analysis showed that SCOPA-AUT subitem 11 (weak stream of urine) was a significant determinant for pRBD.

Regarding lower urinary tract symptoms in PD, although storage symptoms are the most common, voiding dysfunction is also frequent (Chen et al., 2020). Within the “urinary” domain in the present study, item 10 (feeling of residual urine) and item 11 (weak stream of urine), which assessed urine voiding symptoms, showed significant differences between the two groups, as did item 13 (Nocturia). Urinary dysfunctions in PD are thought to be related to nigrostriatal degeneration as well as other central and peripheral lesions in pathology associated with PD (Uchiyama et al., 2011). In PD, the frontal-basal ganglia D1 dopaminergic circuit is affected (Sakakibara et al., 2014). Kitta et al. (2006) demonstrated central involvement of specific brain regions, including periaqueductal gray matter (PAG) and the thalamus, during detrusor overactivity in patients with PD. Urinary functions are also maintained by lower brainstem nuclei, such as the pontine micturition center (PMC) and the pontine continence center. Roy et al. (2019) reported that brainstem damages around the pontine continence center may influence urine storage symptoms in patients with PD. On the other hand, normal micturition is relying on the spino-bulbo-spinal autonomic reflex, which especially involves the midbrain PAG and the PMC (Sakakibara et al., 2014). The PAG is considered a switch center from storage to voiding. The switch mechanism in the PAG is considered to be controlled by higher cerebral structures, such as the hypothalamus and prefrontal cortex, part of which overlap the storage promoting area. Regarding the voiding problem in PD, a correlation between a weak detrusor and the clinical stage was previously suggested (Stocchi et al., 1997), but according to a pressure flow analysis (Sakakibara et al., 2001), half of PD patients showed mild urethral obstruction, and the latter is now considered more critical. Urine voiding problems are a characteristic autonomic impairment more common in MSA than PD (Fanciulli et al., 2019). In our study, more severe voiding symptoms were observed in patients with PD with pRBD compared with those without pRBD, possibly implying that more severe disease-specific pathology in the brainstem nuclei, including PAG and PMC, in patients with PD with pRBD. Postuma et al. (2015a) reported that patients with PD with RBD showed more severe alpha synuclein pathology in the brainstem. In tracking data of 154 PSG-proven RBD patients (Fereshtehnejad et al., 2019), initial manifestations, such as olfaction and color vision, begin to decline over 20 years before conversion to PD, but urinary dysfunction appears relatively late, along with the first motor manifestations, probably at the time when structures including PAG in the midbrain and PMC in the pontine begin to be affected.

Orthostatic hypotension is a frequent cardiovascular symptom of PD. Within the “cardiovascular” domain of the present study, item 14 (symptoms at standing up) showed a significant difference between the PD patients with pRBD and those without pRBD. The disruption of both the sympathetic and parasympathetic structures, susceptible sites in early PD based on Braak staging (Braak et al., 2003), may contribute to the imbalances in blood pressure and heart rate variability. In PD, low baroreflex sensitivity is closely related to orthostatic hypotension, and failure to increase total peripheral resistance due to impaired sympathetic innervation substantially reduces systolic blood pressure (Nakamura et al., 2014). Although there are limited reports of objective assessment of orthostatic hypotension in patients with isolated RBD, Postuma et al. (2008) reported that the presence of RBD in PD was strongly associated with the signs and symptoms of orthostatic hypotension and orthostatic symptom prevalence. In another study of PD patients, there was a significant difference in the presence of orthostatic hypotension among the RBD-complicated group, the subclinical RBD group with RWA on polysomnography, and the normal REM sleep group, with the RBD-complicated group showing more severe orthostatic dysfunction (Nomura et al., 2013). In the evaluation of heart rate variability as an autonomic function, RR variability was lower in RBD patients, and frequency analysis showed a significant decrease in very low frequency, low frequency, and total power (Postuma et al., 2010). Taken together, more severe sympathetic nerve dysfunction may be related to the symptoms of orthostatic hypotension, which is consistent with the results of the present study that cardiac denervation was severe in PD with pRBD. Kim et al. (2016) also reported the close association between RBD and orthostatic hypotension and cardiac sympathetic denervation in patients with relatively early-stage PD, and cardiac sympathetic denervation may be more closely associated with the presence of RBD than with PD (Zitser et al., 2019). Interestingly, the severity of autonomic symptoms may be related to an accelerated rate of phenoconversion in isolated RBD (Li et al., 2017).

The present study has some limitations. First, we lacked a control group. However, we conducted a comprehensive detailed clinical assessment, including cardiac MIBG scintigraphy and olfactory testing. Second, in this study, although the clinical diagnosis of PD was made based on a detailed specialist assessment, a DAT scan could not be performed on all patients due to alcohol allergy or refusal of examination. Third, we used the MMSE as the cognitive assessment. It has been reported that the Montreal Cognitive Assessment was superior to the MMSE as a screening instrument for cognitive decline in PD (Hoops et al., 2009). Fourth, the diagnosis of RBD was based on the RBDSQ-J questionnaire (Miyamoto et al., 2009). Although polysomnography is the gold standard for the diagnosis of RBD, it is not practical to perform polysomnography in all patients with PD. Although RBDSQ-J score cutoff values of 5 or 6 are widely used in clinical studies (Miyamoto et al., 2009; Nomura et al., 2011), we set an RBDSQ-J score of 5 or above to improve sensitivity (Stiasny-Kolster et al., 2007). Fifth, it was previously reported that SCOPA-AUT was insufficient to assess neurologic orthostatic hypotension (Baschieri et al., 2021). We retrospectively rechecked the medical records of all participants; however, only 62 patients were assessed with a head-up tilt test or by orthostatic tests. Eleven (24%) of 45 patients without pRBD and 8 (47%) of 17 patients with pRBD showed an over 20 mmHg drop in systolic blood pressure. Although more patients with pRBD tended to have a systolic pressure drop of 20 mmHg or more (47 vs. 24%), the difference did not reach significance (*P* = 0.085). Sixth, although we excluded the patients who were identified as snorers by interviews with patients and bed partners, the score of PDSS-2 item 15 was statistically different between two groups. The possibility of obstructive sleep apnea potentially interfered the results of present study, such as nocturia and tired/sleepiness, cannot be completely excluded.

## Conclusion

Parkinson’s disease patients with pRBD showed distinct sleep-related symptoms and autonomic symptoms, including visual hallucinations, daytime sleepiness, urine voiding dysfunction, and orthostatic hypotension.

## Data Availability Statement

The original contributions presented in the study are included in the article/supplementary material, further inquiries can be directed to the corresponding author.

## Ethics Statement

The studies involving human participants were reviewed and approved by the Institutional Review Board of Dokkyo Medical University. The patients/participants provided their written informed consent to participate in this study.

## Author Contributions

HF, KO, TS, HS, NN, and KS: conceptualization. HF and KO: data curation. KS: formal analysis and supervision. TS, HS, and NN: investigation and methodology. HF: project administration and writing – original draft. KO, TS, HS, NN, and KS: writing – review and editing. All authors contributed to the article and approved the submitted version.

## Conflict of Interest

The authors declare that the research was conducted in the absence of any commercial or financial relationships that could be construed as a potential conflict of interest.

## Publisher’s Note

All claims expressed in this article are solely those of the authors and do not necessarily represent those of their affiliated organizations, or those of the publisher, the editors and the reviewers. Any product that may be evaluated in this article, or claim that may be made by its manufacturer, is not guaranteed or endorsed by the publisher.
